# The controlling nutritional status score and risk factors associated with malnutrition in patients with acute ischemic stroke

**DOI:** 10.3389/fneur.2023.1067706

**Published:** 2023-03-09

**Authors:** Yuanyuan Chen, Hongyan Yang, Meijuan Lan, Hui Wei, Yanqin Chen

**Affiliations:** Nursing Department, The Second Affiliated Hospital of Zhejiang University School of Medicine, Hangzhou, China

**Keywords:** risk factor, acute stage, ischemic stroke, controlling nutritional status, malnutrition

## Abstract

**Objectives:**

Malnutrition is an independent risk factor for poor outcomes in patients who suffered an acute ischemic stroke (AIS). The controlling nutritional status (CONUT) score can provide information for nutritional management in AIS patients. However, the risk factors associated with the CONUT score have not been established to date. Therefore, in this study, we aimed to investigate the CONUT score of patients with AIS and explore the potential risk factors associated with it.

**Methods:**

We conducted a retrospective review of the data from consecutive AIS patients who were recruited in the CIRCLE study. Within 2 days of admission, we gathered the CONUT score, the Nutritional Risk Screening 2002, the Modified Rankin Scale, the National Institutes of Health Neurological Deficit Score (NIHSS), and demographic data from medical records. We used chi-squared tests to examine admission, and a logistic regression analysis was performed to explore the risk factors associated with CONUT in patients with AIS.

**Results:**

A total of 231 patients with AIS participated in the study, with a mean age of 62.32 ± 13.0 years and a mean NIHSS of 6.77 ± 3.8. Of these patients, 41(17.7%) had hyperlipidemia. In terms of nutritional assessment, 137(59.3%) patients with AIS had high CONUT scores, 86(37.2%) patients with AIS had low or high BMI, and 117(50.6%) patients with AIS had NRS-2002 scores below 3. The chi-squared tests showed that age, NIHSS, body mass index (BMI), and hyperlipidemia were associated with the CONUT score (*P* < 0.05). The logistic regression analysis showed that low NIHSS scores (OR = 0.055 95% CI: 0.003–0.893), younger age (OR = 0.159 95% CI: 0.054–0.469), and hyperlipidemia (OR = 0.303 95% CI: 0.141–0.648) were independently associated with lower CONUT scores (*P* < 0.05), whereas BMI was not found to be independently associated with the CONUT.

**Conclusions:**

More than half of the patients with AIS were at risk of malnutrition, with age and neurological deficits being identified as risk factors for nutritional control. Hyperlipidemia was found to be a protective factor of the CONUT, while NRS-2002 and BMI did not affect the nutritional control in patients with AIS.

## Introduction

It is well-known that nutrition is an independent predictor of outcomes in patients with acute ischemic stroke (AIS) (1). AIS patients with malnutrition show a higher frequency of pneumonia, other infections, and bedsores (2). Malnutrition also indicates poor prognosis, such as prolonged hospital stays and disability-related hospital costs (3). In addition, a previous study suggested that malnutrition may negatively affect functional recovery (4). Patients with AIS are vulnerable to the risk of malnutrition because of dysphagia, impaired consciousness, and the inability to maintain an adequate and healthy diet (5). Thus, ensuring proper nutrition is available to patients with AIS is essential to their recovery.

The early evaluation of nutritional status is crucial for developing appropriate nutrition support for patients with AIS. At hospital admission, the prevalence of malnutrition ranged from 7 to 34% in patients with AIS (6). The wide prevalence range may be attributed to the heterogeneous nutritional assessment methods. The European Society for Clinical Nutrition and Metabolism (ESPEN) has the diagnostic criteria for malnutrition but does not assess the risk of malnutrition (7). Although there is no gold standard for screening the risk of malnutrition, there are several nutritional assessment tools, including the Nutritional Risk Screening 2002 (NRS-2002) (8), the Malnutrition Universal Screening Tool (MUST) (9), the Geriatric Nutritional Risk Index (GNRI) (10), the Prognostic Nutritional Index (PNI) (4), the Subjective Global Assessment (SGA) (11), and anthropometric measurements (12). These tools have been used to evaluate nutritional status in patients with various disorders, but they are not always appropriate for patients with emerging diseases such as stroke because of the difficulties in gathering nutritional information (13). Thus, developing a simple and valid tool to assess the risk of malnutrition is significant for nutrition intervention in patients with AIS.

The controlling nutritional status (CONUT) score was initially proposed by Ignacio de Ulíbarri et al. (14) and is used for hospitalized patients. Then, the CONUT score was used for patients with AIS. The CONUT score is a comprehensive and appropriate tool for assessing nutritional status and a more vital prognostic marker for functional recovery in patients with AIS compared to others (15, 16). Furthermore, the CONUT score has predictive validity for all-cause mortality after 3 months in patients who have suffered a stroke (17). In other words, the CONUT score, as a convenient and cost-effective index, can reflect the nutritional status and functional outcomes of patients with AIS.

However, research on the CONUT score focused on its validity and prediction ability in patients with AIS (18). Considering the fact that malnutrition is relatively common in stroke survivors and could result in serious outcomes, identifying the risk factors for the CONUT in patients with AIS is essential. Therefore, this study aimed to identify potential risk factors that might influence the CONUT score in patients with AIS.

## Methods

### Sample and procedures

In this study, we conducted a retrospective review of the data from consecutive patients who were recruited in the CIRCLE study (19) (ClinicalTrials.gov ID: NCT03702452) between 21 November 2018 and 19 November 2019. The CIRCLE study was designed to verify whether nursing-directed rehabilitation in patients who suffered ischemic strokes can compensate for the lack of professional rehabilitation therapists (20). The inclusion criteria were as follows: (1) patients aged between 18 and 90 years; (2) those having a diagnosis of ischemic stroke by CT or MRI and having met the diagnostic criteria prescribed by the WHO; (3) having an initial ischemic stroke within 7 days with limb dysfunction (muscle strength of the limbs is < 5); (4) those maintaining consciousness (National Institutes of Health Neurological Deficit Score consciousness level 0 or 1); and (5) those who signed an informed consent form. The exclusion criteria were as follows: (1) patients with blood vessels that were recanalized after thrombolysis or thrombectomy; (2) those with cardiopulmonary dysfunction; a history of craniocerebral trauma, fracture trauma, or rheumatoid arthritis; or a physical disability or other diseases that have an impact on the disabled limb; (3) those with cognitive impairment or other mental illness that prevents cooperation with researchers; and (4) those with diseases that affect lymphocyte count. A total of 231 patients were included in this study.

### Ethics approval

Informed consent was obtained from the participants before the study, and the human ethics committee approved the protocols of the Second Affiliated Hospital of Zhejiang University. All clinical investigations were conducted according to the principles expressed in the Helsinki Declaration.

### Evaluation

All the participants were administered the CONUT score, the Nutritional Risk Screening 2002 (NRS-2002), the Modified Rankin Scale (MRS), and the National Institutes of Health Neurological Deficit Score (NIHSS). Demographic data [age, gender, educational background, body mass index (BMI), the limbs' muscles, hypertension, hyperlipidemia, and diabetes] were gathered from medical records within 2 days from admission.

### The controlling nutritional status score

The CONUT scores calculated from the serum albumin concentration, total peripheral lymphocyte count, and total cholesterol concentration are listed in Table 1. The range of the CONUT scores is from 0 to 12; a score of 0 or 1 indicates a normal nutritional status, and higher scores indicate a worse nutritional status (14). According to the original stratification of the CONUT score (normal nutritional status: 0–1; mild malnutrition: 2–4; moderate malnutrition: 5–8; severe malnutrition: 9–12) (21), a CONUT score of 0–4 was used to define the lower risk of malnutrition, and a CONUT score of 5–12 was used to define the higher risk of malnutrition (moderate or severe) in this study. The CONUT score is an effective tool for early detection and continuous control of hospital undernutrition in cases of stroke (18). The samples were collected and analyzed within 2 days of admission.

**Table 1:** Scoring system for the CONUT score.

**Parameter**	**None**	**Light**	**Moderate**	**Severe**
Serum albumin (g/dL)	≥3.5	3.00–3.49	2.50–2.99	< 2.50
Score	0	2	4	6
Total lymphocyte count (/mm^3^)	≥1,600	1,200–1,599	800–1,199	< 800
Score	0	1	2	3
Total cholesterol (mg/dL)	≥180	140–179	100–139	< 100
Score	0	1	2	3

### Nutritional Risk Screening 2002

The NRS-2002 score was calculated from BMI, weight loss over the past 3 months, food intake in recent weeks, and the presence of a fatal disease. Patients with a score of ≥3 were considered at risk of malnutrition (8). The sample was obtained within 2 days of admission.

### Modified Rankin Scale

The modified Rankin Scale (MRS) was used to assess the function of patients who suffered a stroke. The MRS ranged from 0 to 6. The range of 0 indicated a normal function status, and higher ranges indicated a worse function status (22). We also recorded the function result 2 days after admission.

### National Institutes of Health Neurological Deficit Score

The NIHSS assesses the degree of neurological deficits. It consists of 11 parameters, with a maximum score of 42 points. A score of 0 or 1 indicates a normal neurological status, and higher scores suggest a severe neurological deficit (23). In this study, we recorded the neurological deficit result within 2 days of admission for each patient.

### Statistical analysis

Data analysis was conducted using SPSS 24.0. All statistical tests were two-tailed, and an alpha of 0.05 was used to indicate significance. The categorical data were statistical frequencies, including age, gender, education background, muscle of limbs, BMI, NIHSS, MRS, NRS 2002, dysphagia, hypertension, diabetes, and hyperlipidemia, and the chi-squared tests were used to examine the data. Then, a logistic regression analysis with backward stepwise selection (*P* > 0.10 for exclusion) was performed to examine the association between the CONUT score, age, BMI, hyperlipidemia, and the NIHSS.

### Patient and public involvement

Patients and/or the public were not involved in this research's design, conduct, reporting, or dissemination plans.

## Results

### Clinical characteristics of patients with AIS

We administered a questionnaire to 231 patients with AIS. The sample comprised 70.1% male participants with a mean age of 62.32 ± 13.0 years. The mean NIHSS score was 6.77 ± 3.8. The mean MRS score was 3.87 ± 0.4. The mean score of the MRC scale was 2.31 ± 1.7 in the upper limbs and 2.87 ± 1.5 in the lower limbs. Comorbidities consisted of hypertension (68.0%), dysphagia (39.4%), hyperlipidemia (17.7%), and diabetes (26.8%). Regarding nutritional assessment, 137 (59.3%) patients with AIS had high CONUT scores, 86 (37.2%) patients with AIS had low or high BMI, and the NRS-2002 scores of 117 (50.6%) patients with AIS were below 3. The demographic characteristics of the 231 patients with AIS are summarized in Table 2.

**Table 2:** Demographic and clinical information for all participants with CONUT.

**Characteristic**	**Total**	**Low CONUT (*n* = 94)**	**High CONUT (*n* = 137)**	***P***
**Age**				< 0.001^**^
18–45 y	25/231	17/94	8/137	
46–69 y	140/231	61/94	79/137	
>70 y	66/231	16/94	50/137	
Men	162/231	61/94	101/137	0.150
**Education**				0.263
Incomplete elementary school	100/231	36/94	64/137	
Elementary school	73/231	36/94	37/137	
Upper secondary school	43/231	15/94	28/137	
University	15/231	7/94	8/137	
**NHISS**				0.002^*^
0–1	13/231	11/94	2/137	
2–4	69/231	32/94	37/137	
5–14	144/231	50/94	94/137	
15–20	5/231	1/94	4/137	
**MRS**				0.396
3	35/231	17/94	18/137	
4	191/231	76/94	115/137	
5	5/231	1/94	4/137	
**BMI**				0.005^*^
< 18.5	7/231	1/94	6/137	
18.5–24.9	145/231	49/94	96/137	
25–29.9	70/231	38/94	32/137	
30–34.9	9/231	6/94	3/137	
**NRS2002**				0.133
< 3	114/231	52/94	62/137	
≥3	117/231	42/94	75/137	
Dysphagia	91/231	30/94	61/137	0.054
Hypertension	157/231	65/94	92/137	0.749
Diabetes	62/231	24/94	38/137	0.710
Hyperlipidemia	41/231	28/94	13/137	< 0.001^**^
**Muscle strength of upper limb**				0.079
0	59/231	17/94	42/137	
1	25/231	7/94	18/137	
2	22/231	13/94	9/137	
3	43/231	21/94	22/137	
4	74/231	33/94	41/137	
5	8/231	3/94	5/137	
**Muscle strength of lower limb**				0.064
0	31/231	7/94	24/137	
1	18/231	5/94	13/137	
2	21/231	8/94	13/137	
3	61/231	30/94	31/137	
4	80/231	32/94	48/137	
5	20/231	12/94	8/137	

^*^Statistically significant at *p* < 0.05; ^**^statistically significant at *p* < 0.001.

CONUT, controlling nutritional status; NNIHSS, National Institutes of Health Neurological Deficit Score; MRS, Modified Rankin Scale; NRS-2002, Nutritional Risk Screening 2002.

### Risk factors for the CONUT in patients with AIS

Table 2 shows that age, NIHSS, BMI, and hyperlipidemia were associated with the CONUT score (*P* < 0.05), according to the results of the univariate analysis. A logistic regression analysis revealed that those patients with AIS who had low NIHSS scores (0–1) were independently associated with lower CONUT scores compared with those with high NIHSS scores (OR = 0.055 95% CI: 0.003–0.893). Younger age (18–44 years) was independently associated with lower CONUT scores than older age (OR = 0.159 95% CI: 0.054–0.469). AIS patients with hyperlipidemia were independently associated with lower CONUT scores (OR = 0.303 95% CI: 0.141–0.648). BMI was not shown to be independently associated with the CONUT scores (Table 3).

**Table 3:** Logistic regression analysis of CONUT.

**Factor**	***B***	**SE**	**Wald**	***P*-value**	**OR**	**95% CI**
**NHISS (15–20)**						
NHISS (0–1)	−2.906	1.425	4.158	0.041^*^	0.055	0.003–0.893
NHISS (2–4)	−0.849	1.221	0.483	0.487	0.428	0.039–4.686
NHISS (5–14)	−0.469	1.205	0.152	0.697	0.625	0.059–6.633
**Age (above 70 y)**						
Age (18–45 y)	−1.839	0.553	11.078	0.001^*^	0.159	0.054–0.469
Age (46−69 y)	−0.945	0.366	6.662	0.10	0.389	0.190–0.947
Hyperlipidemia	−1.195	0.388	9.469	0.002^*^	0.303	0.141–0.648
Constant	2.103	1.206	3.043	0.081	8.189	N/A

^*^Statistically significant at *p* < 0.05.

CONUT, Controlling Nutritional Status; NNIHSS, National Institutes of Health Neurological Deficit Score.

## Discussion

In this study, the main findings are as follows: (1) More than half of the patients with AIS are at risk for malnutrition or poor nutritional control; (2) older age is independently associated with poor nutritional control; (3) the degree of neurological deficit affects the nutritional control of patients with AIS (severe neurological deficits indicate bad nutritional status); (4) hyperlipidemia is also an independent factor of nutritional status in patients with AIS; and (5) the nutritional assessment of the NRS-2002 and BMI may not affect the nutritional control in patients with AIS.

The CONUT score as a nutritional risk assessment tool is easy to obtain from biochemical parameters, which reflect the risk of malnutrition because of the comprehensive assessment of nutritional status. This study found that more than half of the patients with AIS are under poor nutritional control. This result is similar to the study by Naito et al. (16). In other words, patients who suffered a stroke are likely to experience nutritional problems in the acute stage. It is essential to evaluate the nutritional status of patients with AIS because malnutrition may worsen clinical outcomes (17). Exploring the risk factors for nutritional status can help prevent malnutrition.

In this study, older age was a poor prognosticator for nutritional control. For older adults, natural age-related changes could increase the risk of malnutrition (24). If they suffered acute stroke events, increased protein requirements, alterations to appetite, and declining sensory function could worsen their condition. A previous study showed that advanced age was the main risk factor for malnutrition in patients who suffered a stroke and that it affects recovery (25). In other words, the risk of malnutrition is higher in older patients with AIS, leading to poor outcomes. This study also showed that the risk of malnutrition increases with age.

The NIHSS is a scale that reflects neurological deficits. This study showed that the NIHSS is the independent factor of the CONUT score. In brief, the degree of neurological deficit affects the nutritional control of patients with AIS. AIS patients with critical neurological deficits often have reduced consciousness levels, dysphagia, facial or arm weakness, reduced mobility, cognitive impairments, and poor oral hygiene (26). In the acute stage of a stroke, dysphagia is a significant factor in developing malnutrition, which occurs in 30–50% of patients (27). The disturbance of consciousness may cause patients to not be fed in time after they suffer a stroke, leading to malnutrition. The presence of depression, cognitive impairments, and language deficits could hinder effective communication about food preferences and satiety, leading to malnutrition, particularly with regard to protein intake (28). Moreover, the paralysis of dextromanuality and weakness affects patients' food intake, leading to a premature suspension of feeding (29). In summary, the neurological deficit is an important factor of CONUT in patients with AIS.

In this study, we found an interesting result: AIS patients with hyperlipidemia were associated with lower CONUT scores. In other words, hyperlipidemia may result in a low risk of malnutrition. Cholesterol, an item of the CONUT score, is a part of blood fat, which might be the main reason why hyperlipidemia affects malnutrition. Since hyperlipidemia is a risk factor for cardiovascular and cerebrovascular diseases, maintaining an average blood fat level is crucial for health (30).

In contrast, BMI was not found to be independently associated with the CONUT scores. The most likely explanation is that only seven patients with AIS in the study had a low BMI. Moreover, the risk of malnutrition in the acute stage may not immediately lead to a low BMI soon, meaning that BMI does not reflect current changes in nutrition but is an indicator of malnutrition.

### Implications for practice

This study highlights the risk factors associated with malnutrition in patients with AIS and emphasizes the need for early nutritional evaluation and intervention. Close attention should be paid to older patients, especially those with severe neurological deficits. Effective nutritional intervention measures should be developed to decrease the occurrence of malnutrition.

### Limitations

This study has some limitations. First, the CONUT score for the chronic stage was not collected; therefore, it was impossible to compare the nutritional control status at different stages. Second, the sample size may be more significant to obtain more stable results. Finally, only one hospital's patients with AIS were included, which may create a representational bias, that is, the representation of all hospitalized individuals was missing, and the study findings might not apply to all patients with AIS.

## Conclusion

The risk of malnutrition is high among patients with AIS. The CONUT score can reflect malnutrition. The significant risk factors associated with the CONUT score are older age, severe neurological deficits, and hyperlipidemia. The NRS-2002 and BMI may not be effective screening tools for identifying the risk of malnutrition in the acute stage of patients with stroke.

## Data availability statement

The raw data supporting the conclusions of this article will be made available by the authors, without undue reservation.

## Ethics statement

The Human Ethics Committee approved the protocols of The Second Affiliated Hospital of Zhejiang University. The patients/participants provided their written informed consent to participate in this study.

## Author contributions

YuC and ML provided critical feedback, edits to drafts of the paper, and managed subsequent revisions. HY contributed to the design of the study and facilitated data acquisition. YaC and HW contributed to the data acquisition and analysis. All authors read and approved the final manuscript.
